# Pneumothorax and pulmonary hemorrhage after C-arm cone-beam computed tomography-guided percutaneous transthoracic lung biopsy: incidence, clinical significance, and correlation

**DOI:** 10.1186/s12890-023-02822-9

**Published:** 2024-01-13

**Authors:** Yanjie Yang, Jingqin Ma, Zhijie Peng, Xin Zhou, Nan Du, Wen Zhang, Zhiping Yan

**Affiliations:** 1 Department of Interventional Radiology, Zhongshan Hospital, Fudan University, Shanghai, China; 2 Shanghai Institute of Medical Imaging, Shanghai, China

**Keywords:** Percutaneous transthoracic lung biopsy, C-arm cone-beam CT, Pulmonary hemorrhage, Pneumothorax

## Abstract

**Objective:**

This study aimed to assess the incidence and clinical significance of pneumothorax (PTX) and pulmonary hemorrhage (PH) after percutaneous transthoracic lung biopsy (PTLB) guided by C-arm cone-beam computed tomography (CBCT). Furthermore, this study aimed to examine the relationships between PTX and PH with demographics, clinical characteristics, imaging, and PTLB parameters.

**Methods:**

A retrospective analysis was conducted on 192 patients who underwent PTLB at our hospital between January 2019 and October 2022. Incidences of PTX and PH were recorded. PTX was considered clinically significant if treated with chest tube insertion (CTI), and PH if treated with bronchoscopes or endovascular treatments. The various factors on PTX and PH were analyzed using the Chi-squared test and Student t-test. Logistic regression analyses were then used to determine these factors on the correlation to develop PTX and PH.

**Results:**

PTX occurred in 67/192 cases (34.9%); CTI was required in 5/67 (7.5%). PH occurred in 63/192 cases (32.8%) and none of these cases required bronchoscopes or endovascular treatments. Lesion diameter (OR_PTX_ = 0.822; OR_PH_ = 0.785), presence of pulmonary emphysema (OR_PH_ = 2.148), the number of samples (OR_PH_ = 1.834), the use of gelfoam (OR_PTX_ = 0.474; OR_PH_ = 0.341) and ablation (OR_PTX_ = 2.351; OR_PH_ = 3.443) showed statistically significant correlation to PTX and PH.

**Conclusions:**

CBCT-guided PTLB is a safe and effective method for performing lung biopsies. The use of gelfoam has been shown to reduce the occurrence of PTX and PH. However, caution should be exercised when combining radiofrequency ablation with PTLB, as it may increase the risk of PTX and PH.

## Introduction

Lung cancer remains the leading cause of cancer-related deaths globally [1]. Achieving early detection of lung cancer through screening represents a significant milestone in enhancing prognosis [2]. Chest X-ray is a routine radiologic examination in clinical practice [3]. With the advancement of imaging technology, the adoption of low-dose computed tomography (CT) for lung cancer screening in high-risk individuals holds the potential for detecting lung cancer at an early stage, with the potential to reduce associated mortality rates [4]. When a potentially malignant nodule is identified, obtaining tissue samples through biopsy is included as part of the clinical evaluation process [5].

Percutaneous transthoracic lung biopsy (PTLB) is a dependable approach for the diagnosis of pulmonary nodules [6]. Conventional computed tomography (CCT)-guided PTLB is commonly used due to its widespread availability and high diagnostic accuracy [7, 8]. However, the limitation of CCT-guided PTLB is the lack of real-time visualization during needle insertion. This can increase the procedure time and radiation doses, particularly in cases where deep pulmonary nodules require oblique needle angles to avoid major vessels, ribs, or airways, or in older patients who have difficulty holding their breath [9]. C-arm cone-beam computed tomography (CBCT) combines a C-arm gantry with cone-beam X-ray tube and flat panel detectors to provide high-resolution imaging along with the real-time needle guiding capability of fluoroscopic systems [10, 11]. Utilizing path-planning software and the rotational ability of a C-arm, CBCT empowers operators to approach challenging lesions with greater confidence [12–14].

In addition to its real-time needle guiding capability, CBCT-guided PTLB is considered a safe procedure with pneumothorax (PTX) and pulmonary hemorrhage (PH) being the main procedural complications [15]. Recent studies have demonstrated comparable incidences of PTX and PH when utilizing CBCT guidance and CCT guidance [16–22]. Several studies reported risk factors for PTX and PH such as old age, presence of pulmonary emphysema, lower lobar location, small target size, and deep location [16, 17, 19, 23]. However, iatrogenic PTX is self-limiting in most cases, and only in a very small percentage, it necessitates chest tube insertion (CTI) [17]. Similarly, PH is generally asymptomatic, or it may clinically manifest as light hemoptysis [17]. Massive bleeding is exceptional, but it is a serious condition that may require endobronchial procedures or endovascular embolization [24].

Thus, the purpose of our study was to evaluate the incidence and clinical significance of PTX and PH after CBCT-guided PTLB and to test correlations of PTX and PH with demographics, clinical characteristics, imaging, and PTLB parameters.

## Materials and methods

### Study population and patient selection

**Fig. 1:**
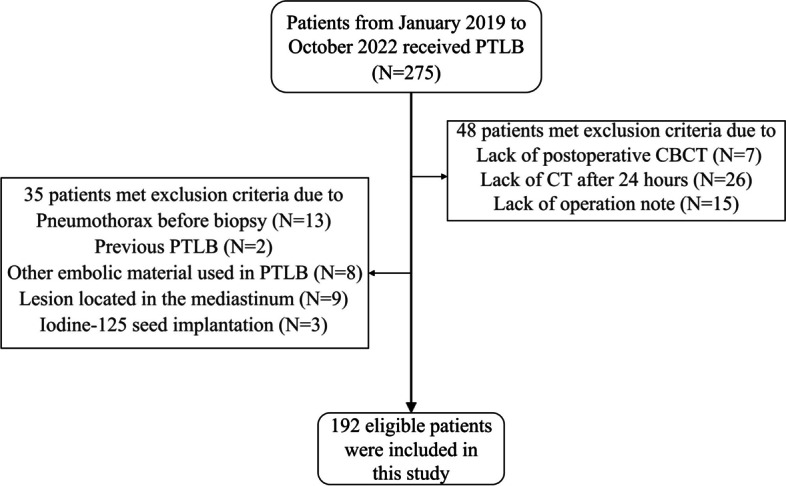
Flow chart showing the enrolled patients. PTLB percutaneous transthoracic lung biopsy, CBCT C-arm cone-beam computed tomography, pneumothorax (PTX), pulmonary hemorrhage (PH)

From January 2019 to October 2022, 275 consecutive patients who received CBCT-guided PTLB were retrospectively included in this study. The following exclusion criteria were applied: (1) Patients with PTX or PH identified during pre-procedural imaging; (2) Patients with a history of PTLB; (3) Patients with target lesions located in the mediastinum, pleura, or chest wall; and (4) Patients with incomplete bioptic records or imaging data. Eighty-three patients were excluded, and thus, a final study population of 192 patients was included (Fig. 1). The Institutional Review Board approved this study.

### Pre- procedural preparation

Patients were subjected to either plain or enhanced CT of the chest, which was conducted within 1 week before the PTLB. Any regular intake of anticoagulants or platelet inhibitors by patients was stopped for at least 3 days before the procedure. Patients were given breathing instructions and advised to maintain consistent breathing during the procedure. Before the PTLB, patients were fully informed about the necessity and possible risks associated with the biopsy procedure, and they were required to provide their written informed consent.

### Overview of PTLB procedure

The puncture procedure was performed by three senior interventional radiologists with over a decade of experience in puncture biopsy. All PTLBs were carried out under local anesthesia with the help of a CBCT virtual navigation guidance system. A coaxial cutting needle technique was used, involving an 18-gauge cutting needle and a 17-gauge biopsy coaxial cannula. Patients were positioned in either the supine or prone position, depending on their lesion location and the presence of ribs or large blood vessels. Before the biopsy, a pre-procedural CBCT scan was conducted of the entire lung to identify the safest and most accessible route to the target nodule(s), while avoiding obstacles and minimizing pleural contact and needle travel distance through the lung parenchyma. To reduce needle course complexity and improve accuracy, needle angulation was kept to the vertical plane of rotation during CBCT procedures. Following PTLB, a strip of sample measuring approximately 1 to 2 cm in diameter and 1.2 mm in width was obtained and immediately fixed in 10% formalin.

For patients with a high suspicion of malignant lung nodules (e.g., ground-glass nodules larger than 1 cm) on pre-procedural CT or those with clinical features consistent with malignancy (e.g., concurrent hepatocellular carcinoma), we use a 17-gauge radiofrequency ablation (RFA) coaxial cannula instead of a 17-gauge biopsy coaxial cannula during the PTLB procedure. After the PTLB, the cutting needle was retrieved, and an RFA needle was inserted through the coaxial cannula that remained in place to perform RFA therapy. Besides, some patients were provided with a gelfoam slurry (created from an absorbable gelatin sponge) to embolize the needle tract following the procedure. The specific procedure is as follows: The 1000-1200 μm gelfoam is placed into a 10-ml syringe. A three-way stopcock was attached and another 10-ml syringe with 10 ml iodinated contrast. The mixture was rapidly agitated between the syringes until the mixture appeared homogenous. After withdrawing the biopsy or RFA needle, slowly remove the coaxial cannula while simultaneously injecting approximately 2-3 ml of the slurry through the coaxial cannula to embolize the needle tract, ensuring this procedure is performed under fluoroscopy.

Post-procedural CBCT images were taken to identify any procedure-related complications. If the post-procedural CBCT indicates the presence of PTX or PH, the patient should undergo evaluation by an interventional radiologist and receive appropriate treatment. After the PTLB, patients were transferred to the ward and monitored for 24 hours. A follow-up chest CT scan was conducted after 24 hours to detect any delayed complications.

### Data collection

The retrospective collection of data for each study participant involved gathering all pertinent demographic, clinical characteristics, imaging, and PTLB parameters. The CT images of each patient were analyzed by two independent readers, including the PTLB operator and an attending-level radiologist. The size of the nodules was determined by measuring the longest diameter of the lesion. The nodules were categorized based on their features, including solid, ground-glass, and cavitary. The presence of pulmonary emphysema was defined as disrupted lung vasculature and parenchyma with low attenuation occupying any lung zone (at least trace) at chest CT [25]. Patients with smoking history were categorized as individuals who have smoked a minimum of 30 packs per year and either currently smoked or have ceased smoking within the last 15 years [26].

**Fig. 2:**
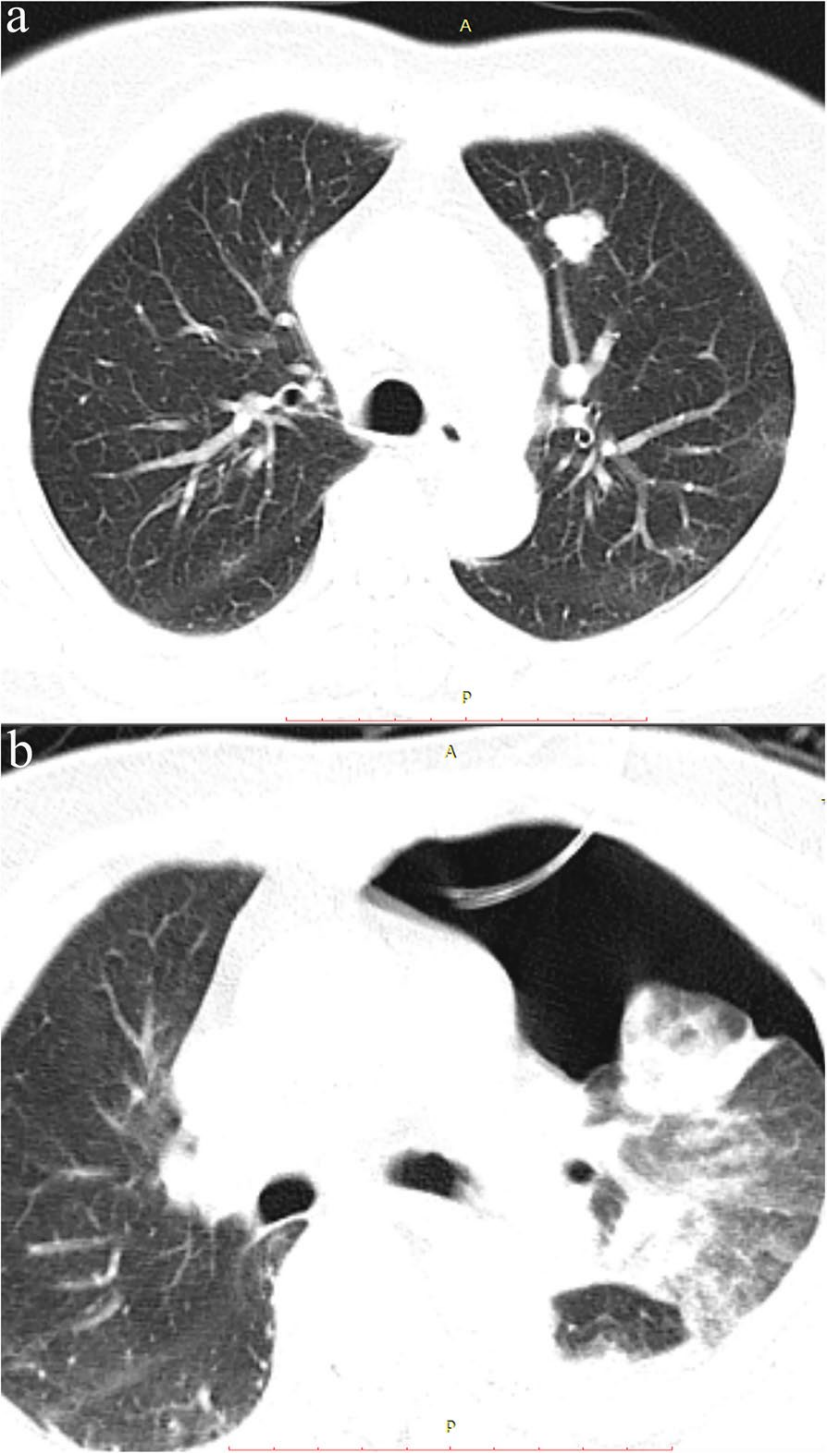
**a** Pre-procedure CT: A 79-year-old man with a history of hepatectomy for hepatocellular carcinoma 2 years ago presented with a 24-mm solid pulmonary lesion in the left lower lobe. **b** Following the combined procedure of percutaneous transthoracic lung biopsy (PTLB) and radiofrequency ablation (RFA), the patient experienced a severe pneumothorax, which necessitated chest tube insertion (CTI). The chest tube was left in place for drainage purposes for 2 days

**Fig. 3:**
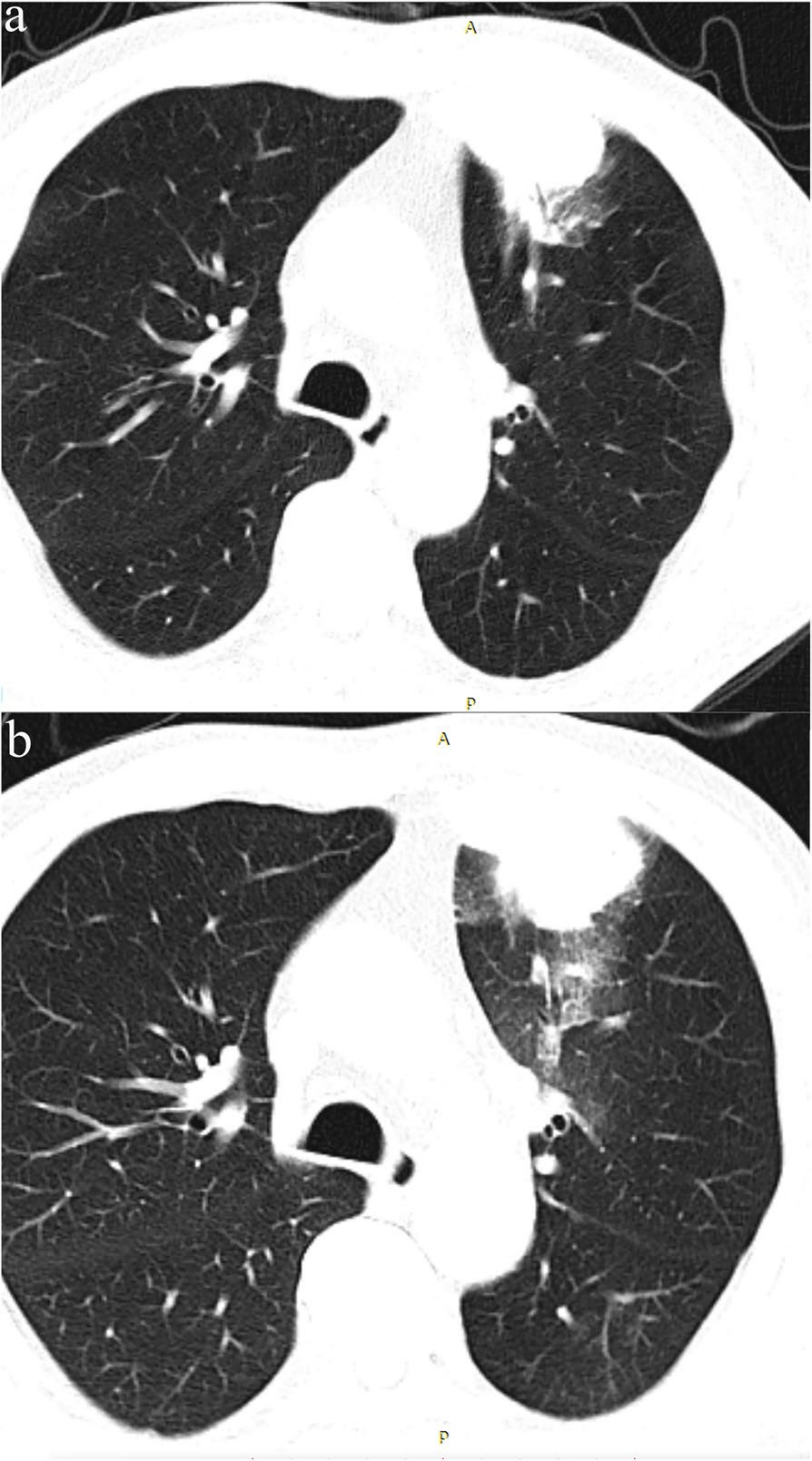
**a** Pre-procedure CT: A 69-year-old man with a 29-mm solid pulmonary lesion in the left lower lobe. **b** On the post-procedural CT scan, a ground-glass opacity was observed in the pulmonary parenchyma. However, the patient did not experience any episodes of hemoptysis

Both PTX and PH are defined based on imaging. The definition of PTX was based on the presence of air in the pleural space and classified according to the timing of appearance: immediate PTX observed on CBCT following PTLB and delayed PTX observed on CT plain scan 24 hours after PTLB. Clinically significant PTX was defined as the occurrence of severe respiratory or circulatory dysfunction that necessitated the insertion of a chest tube for treatment (Fig. 2). PH was defined as the presence of ground-glass opacity in the pulmonary parenchyma, which occurs due to the filling of alveolar spaces with blood (Fig. 3). PH was categorized into four groups: asymptomatic, mild hemoptysis (blood loss less than 100 ml in 24 hours), moderate hemoptysis (blood loss between 100 ml and 500 ml in 24 hours), and severe hemoptysis (blood loss exceeding 500 ml in 24 hours). Clinically significant PH was defined as the need for invasive medical interventions such as bronchoscopes or endovascular treatments to achieve hemostasis.

### Statistical methods

Statistical analysis was performed with SPSS software (version 27.0; SPSS, Chicago, IL). To identify significant factors, subgroup analysis was performed using Student’s *t*-test for continuous variables and Pearson’s chi-squared test for categorical data. Due to the limited sample size, Fisher’s exact test was used. Logistic regression analyses were then used to further determine the effects of evaluated parameters on the likelihood of developing PTX and PH. The results are reported in terms of odd ratios (OR), with their 95% confidence intervals. A *p*-value less than 5% (*p* < 0.05) was considered to be statistically significant.

## Results

### Baseline clinical characteristics and PTLB parameters

The demographic and clinical characteristics of patients and the imaging and procedural parameters are summarized in Table 1. A total of 192 patients underwent PTLB (males 129, 67.2%) with a mean age of 62.1 ± 13.4 years. Of all patients, 29 (15.1%) had a history of smoking, and 47 (24.5%) were diagnosed with pulmonary emphysema. Among those with pulmonary emphysema, 16 patients (34.0%) later experienced post-procedural PTX. The mean diameter of lung lesions was found to be 3.40 ± 2.20 cm, with 51 patients (26.6%) exhibiting lesions in the left upper lobe. Solid nodules were present in 164 of the patients (85.4%), while ground glass and cavity nodules were observed in 22 and 6 patients (11.5 and 3.1%), respectively. During the PTLB procedure, 43 patients (22.4%) proceeded to undergo RFA after PTLB. Gelfoam was utilized to seal the puncture tract in 77 patients (40.1%) following the PTLB procedure.

**Table 1:** Baseline characteristics of all included patients

Characteristics (*n* = 192)	
Sex	
Male	129(67.2%)
Female	63(32.8%)
Age (years)	
Mean ± SD	62.1 ± 13.4
Range	18 ~ 86
Smoking history	
Yes	29(15.1%)
No	163(84.9%)
Pulmonary emphysema	
Yes	47(24.5%)
No	145(75.5%)
Lesion diameter (cm)	
Mean ± SD	3.4 ± 2.2
Range	0.6 ~ 13.8
Lesion feature	
Solid	164(85.4%)
Ground glass	22(11.5%)
Cavity	6(3.1%)
Lesion location	
Right upper lobe	39(20.3%)
Right middle lobe	12(6.3%)
Right lower lobe	49(25.5%)
Left upper lobe	51(26.6%)
Left lower lobe	41(21.4%)
Patient position	
Supine	72(37.5%)
Prone	120(62.5%)
Gelfoam	
Yes	77(40.1%)
No	115(59.9%)
Pathology results	
Benign	48(25.0%)
Malignant	141(73.4%)
Insufficient material	3(1.6%)
Radiofrequency ablation	
Yes	43(22.4%)
No	149(77.6%)
The number of samples extracted	
1	69(35.9%)
2	96(50.0%)
3	21(10.9%)
4	6(3.1%)

Among the pathological biopsies conducted after PTLB, 3 patients (1.6%) were deemed inconclusive due to insufficient material for diagnosis. Out of the 192 patients, 141 (73.4%) were diagnosed with malignant tumors, with 113 patients identified with primary lung cancer and 28 patients with metastatic lesions. Additionally, 48 patients (25.0%) were determined to be benign.

### Complication

PTX was observed in 67 patients (34.9%). Among these patients, immediate PTX occurred in 42 patients (62.7%), while 25 patients (37.3%) experienced PTX within 24 hours. The majority of PTX were self-limiting and resolved spontaneously, as CTI was required in 5 of 67 patients (7.5%). The mean duration of catheter placement was 2.6 ± 0.9 days (Table 2). Due to the limited number of patients requiring CTI, regression analysis examining the factors influencing CTI was not conducted in our study. PH occurred in 63 patients (32.8%). Among these patients, 39 patients (61.9%) were asymptomatic, while 15 patients (23.8%) had mild hemoptysis without the need for any medical intervention. Although 9 patients (14.3%) experienced moderate hemoptysis, these patients showed improvement with appropriate hemostatic drug treatment. None of the patients experienced severe hemoptysis that necessitated invasive medical interventions (Table 2).

**Table 2:** Incidence and types of pneumothorax and pulmonary hemorrhage

Pneumothorax	Incidence	Chest tube insertion
Total	67/192 (34.9%)	5/67 (7.5%)
Immediate CBCT scan	42/67 (62.7%)	2/5 (40%)
24 h on CT scan	25/67 (37.3%)	3/5 (60%)
Pulmonary hemorrhage	Incidence	Bronchoscopes or endovascular treatments
Total	63/192 (32.8%)	0/63 (0%)
Asymptomatic	39/63 (61.9%)	
Mild hemoptysis	15/63 (23.8%)	
Moderate hemoptysis	9/63 (14.3%)	
Severe hemoptysis	0/63 (0%)	

### Subgroup analysis

The outcomes of the subgroup analysis on PTX and PH are presented in Table 3 and Table 4. The analysis revealed that the incidence of PTX was associated with lesion diameter, the use of gelfoam, and RFA (*P* < 0.05). PH was found to be associated with the presence of pulmonary emphysema, lesion diameter, the use of gelfoam, RFA, and the number of samples (*P* < 0.05).

**Table 3:** Subgroup analysis of pneumothorax

Influence factor	Pneumothorax		*t*/c^*2*^,*P*
	Yes(*n* = 67)	No(*n* = 125)	
Sex (Male/female)	45/22	84/41	0.000, 0.996
Age (years)	61.8 ± 15.0	62.3 ± 12.6	0.213, 0.831
Smoking history (Yes/No)	11/56	18/107	0.139, 0.710
Pulmonary emphysema (Yes/No)	16/51	31/94	0.020, 0.888
Lesion diameter	2.8 ± 1.6	3.7 ± 2.4	3.125, 0.002
Lesion feature			
Solid	59	105	2.167, 0.352
Ground glass	5	17	
Cavity	3	3	
Lesion location			
Right upper lobe	16	23	5.549, 0.234
Right middle lobe	3	9	
Right lower lobe	21	28	
Left upper lobe	18	33	
Left lower lobe	9	32	
Patient position (Supine/Prone)	25/42	47/78	0.002, 0.969
Gelfoam (Yes/No)	18/49	59/66	7.509, 0.006
Pathology results(Benign/Malignant/ Insufficient material)	19/46/2	29/95/1	2.228, 0.310
Radiofrequency ablation (Yes/No)	23/44	20/105	8.431, 0.004
The number of samples			
1	28	41	1.668, 0.647
2	31	65	
3	6	15	
4	2	4

**Table 4:** Subgroup analysis of pulmonary hemorrhage

Influence factor	Pulmonary hemorrhage		*t*/c^*2*^,*P*
	Yes(*n* = 63)	No(*n* = 129)	
Sex (Male/female)	45/18	84/45	0.765, 0.382
Age (years)	62.0 ± 14.5	62.2 ± 13.0	0.098, 0.922
Smoking history (Yes/No)	12/51	17/112	1.137, 0.286
Pulmonary emphysema (Yes/No)	21/42	26/103	3.976, 0.046
Lesion diameter	2.7 ± 1.7	3.7 ± 2.3	3.567, 0.001
Lesion feature			
Solid	55	109	1.904, 0.393
Ground glass	5	17	
Cavity	3	3	
Lesion location			
Right upper lobe	18	21	5.094, 0.277
Right middle lobe	5	7	
Right lower lobe	13	36	
Left upper lobe	16	35	
Left lower lobe	11	30	
Patient position (Supine/Prone)	24/39	48/81	0.014, 0.905
Gelfoam (Yes/No)	15/48	62/67	10.365, 0.001
Pathology results(Benign/Malignant/ Insufficient material)	17/45/1	31/96/2	0.431, 0.876
Radiofrequency ablation (Yes/No)	20/43	20/109	10.744, 0.001
The number of samples			
1	18	51	8.048, 0.038
2	34	62	
3	6	15	
4	5	1	

### Logistic regression

Significant factors for subgroup analysis were incorporated into the logistic regression analyses. Table 5 displays the findings of the logistic regression analysis conducted to assess the impact of parameters on PTX and PH. Lesion diameter (OR = 0.822, per centimeter), the use of gelfoam (OR = 0.474), and RFA therapy (OR = 2.351) were identified as potential influencing factors for PTX. Lesion diameter (OR = 0.785, per centimeter), the use of gelfoam (OR = 0.341), RFA therapy (OR = 3.443), the presence of pulmonary emphysema (OR = 2.148), and the number of samples (OR = 1.834, per sample) were identified as potential influencing factors for PH.

**Table 5:** Logistic regression analyses of pneumothorax and pulmonary hemorrhage

	*P*	OR	CI
Pneumothorax			
Lesion diameter	0.025	0.822	0.692–0.975
Gelfoam	0.028	0.474	0.244–0.923
Radiofrequency ablation	0.020	2.351	1.147–4.820
Pulmonary hemorrhage			
Lesion diameter	0.014	0.785	0.648–0.952
Gelfoam	0.005	0.341	0.162–0.718
Pulmonary emphysema	0.047	2.148	1.009–4.572
The number of samples	0.013	1.834	1.138–2.956
Radiofrequency ablation	0.002	3.443	1.555–7.627

## Discussion

CBCT is a technology that integrates flat detectors with cone-beam CT within an angiography-interventional C-arm, demonstrating sufficient bone and soft-tissue resolution in preclinical investigations to aid minimally invasive head and neck surgery [27]. The primary advantage of using CBCT systems for percutaneous needle procedures is the real-time imaging during needle insertion, which simplifies needle path planning and improves the accuracy of reaching target lesions. Furthermore, the use of flat-panel CBCT systems has the potential to reduce procedure times and, subsequently, radiation doses for patients. The objective of our study was to analyze the incidence, clinical significance, and correlation of two complications, PTX and PH.

PTX and PH have a tangible impact on patient management and discomfort levels. Mild cases of PTX and PH can contribute to prolonged hospital stays for patients, while severe cases of PTX and PH can lead to respiratory and circulatory system disorders, posing a significant risk to patient safety. The incidence of PTX and PH in this study were similar (34.9 and 32.8%, respectively), consistent with previous literature on CT-guided procedures [28–33]. Conservative treatment is effective in improving the vast majority of PTX and PH cases. The requirement for CTI was observed in only 7.5% of cases, which aligns with the incidence previously reported, ranging from 2.4 to 15% [23, 34, 35]. None of those with PH required bronchoscopes or endovascular treatments. Furthermore, no major complications, such as liver or spleen injury, air embolism, or mortality, were observed in our study. These findings suggest that CBCT-guided PTLB is a relatively safe procedure, characterized by a low incidence of clinically significant complications and no requirement for a prolonged hospital stay in the majority of patients.

While considering many factors such as demographic, clinical characteristics, imaging, and PTLB parameters, only a handful of them showed statistically significant results. The most significant finding is the impact of gelfoam on reducing the incidence of PTX and PH (OR value decreased by 56.8 and 69% respectively). This finding holds clinical significance due to the accessibility and simplicity of using gelfoam in medical practice. Since the initial performance of PTLB, interventional radiologists have explored different techniques to reduce the risk of complications [36–38]. Among these methods, the closure of the needle tract using various embolizing materials has received considerable research attention. The injection of gelfoam leads to its expansion within the needle tract, creating a dense filling that conforms to the shape of the tract. This effectively prevents bleeding and the entry of intrapulmonary air into the pleural cavity through the puncture tract, as well as pleural rupture. Renier et al. created a slurry by cutting 15 pieces of a 2 × 6 cm absorbable gelatin sponge into roughly equal sizes and mixing them with 2 ml of saline [39]. They successfully reduced the incidence of PTX and CTI by sealing the puncture tract with this slurry. In contrast to previous studies, our study utilized smaller gelatin sponge particles with a size range of 1000–1200 μm. This approach not only reduced the preparation time of the slurry but also allowed for a more dense sealing of the puncture tract.

A larger diameter demonstrated a protective effect against both PTX and PH, with OR of 0.822 and 0.785 per centimeter, respectively. On the contrary, an increased number of samples extracted during PTLB was associated with a higher likelihood of developing PH, with a corresponding increase in the OR (83.4% for each additional piece of samples removed). These two observations are in line with previous findings and highlight the correlation between the level of technical complexity of the PTLB procedure and the incidence of complications [17, 20]. Biopsying large lesions is easier and requires less time for needle placement within the parenchyma, resulting in a lower probability of developing procedural complications. In contrast, obtaining samples from repeated punctures can be challenging, require a longer procedure time, and have a higher risk of complications.

Our study reveals that RFA therapy performed after PTLB is associated with an increased risk of both PTX and PH (OR_PTX_ = 2.351; OR_PH_ = 3.443). Previous studies have shown that performing PTLB and RFA therapy in the same procedure can avoid multiple punctures [40]. Therefore, in our study, patients with a high suspicion of malignant lung nodules underwent RFA following PTLB. Schneider et al. reported in their study that performing a biopsy immediately before RFA may result in PH or PTX, which can compromise the accuracy of subsequent RFA needle placement by blurring or displacing the tumor [41]. This is because an additional puncture was necessary during the biopsy due to the unavailability of a suitable guiding cannula. However, in our study, no additional punctures were required during the establishment of the puncture tract due to the use of a multifunctional coaxial cannula. Following the PTLB, only the cutting needle was retrieved while the coaxial cannula remained in place. This method not only guarantees the accuracy of pathological results but also reduces the number of required punctures. Izaaryene et al. conducted pathological investigations after radiofrequency ablation of the lung in pigs and observed distinct needle tracts compared to a simple biopsy. They identified unique histological changes within the ablation tracts, which were likely attributable to thermal effects [42]. This study indicates that the needle tract created after RFA may tend to remain open for an extended period compared to biopsy alone, potentially leading to PTX and PH.

In addition, our study discovered that the occurrence of pulmonary emphysema increases the risk of PH. This finding is in line with previous research [17]. A previous study has demonstrated a significant correlation between pulmonary emphysema and PH and proposed that the heightened risk could be a result of pulmonary hypertension [43]. An alternative explanation is that in patients with pulmonary emphysema, the destruction of air-space walls beyond the terminal bronchioles could create additional space for the PH to expand, leading to an increased risk of complications [44]. In conclusion, heightened vigilance is necessary when conducting biopsies on patients with pulmonary emphysema due to the increased risk of complications.

Several limitations of this study should be acknowledged. Firstly, the study is retrospective. Due to the limitations of the available procedure records, we were unable to further explore several influencing factors, such as the distance between the puncture site and the lesion. Secondly, it is a single-center study, which may limit the generalizability of the results. Thirdly, the limited number of patients requiring CTI, bronchoscopes, or endovascular treatments restricted our ability to conduct a comprehensive analysis of the factors influencing the rate of these interventions.

##  Conclusion

In conclusion, CBCT-guided PTLB is a reliable technique that is widely used in the diagnosis of pulmonary lesions. Nonetheless, akin to CT-guided PTLB, PTX and PH persist as prominent complications. To reduce these complications, this study introduces an innovative and feasible approach—using gelfoam to embolize the puncture tract. This method has demonstrated a noteworthy reduction in the complication rate.

## Data Availability

The datasets used and/or analysed during the current study available from the corresponding author on reasonable request.

## Abbreviations

CT: Computed tomography
PTLB: Percutaneous transthoracic lung biopsy
CCT: Conventional computed tomography
CBCT: C-arm cone-beam computed tomography
PTX: Pneumothorax
PH: Pulmonary hemorrhage
CTI: Chest tube insertion
RFA: Radiofrequency ablation
OR: Odd ratios
